# Mining care trajectories using health administrative information systems: the use of state sequence analysis to assess disparities in prenatal care consumption

**DOI:** 10.1186/s12913-015-0857-5

**Published:** 2015-05-15

**Authors:** Nolwenn Le Meur, Fei Gao, Sahar Bayat

**Affiliations:** Department of Epidemiology and Biostatistics, French School of Public Health (EHESP), Sorbonne Paris Cite, France; EHESP, EA 7348 MOS Management des organisations en santé, EHESP, Avenue du Professeur Leon Bernard, 35043 Rennes, France; Inserm, UMR IRSET Institut de recherche sur la santé l’environnement et le travail, 1085 Rennes, France

**Keywords:** Medico-administrative databases, Care trajectories, State sequence data analysis, Pregnancy, Disparities

## Abstract

**Background:**

Pregnant women are a vulnerable population. Although regular follow-ups are recommended during pregnancy, not all pregnant women seek care. This pilot study wanted to assess whether the integration of data from administrative health information systems and socio-economic features allows identifying disparities in prenatal care trajectories.

**Methods:**

Prenatal care trajectories were extracted from the permanent sample of the French health insurance information system linked to the hospital discharge information system. The records of 2518 women who gave birth without complications in France in 2009 were analyzed. State sequence data analysis was performed to identify homogeneous groups of prenatal care trajectories. Socio-economic data were used to characterize their living environment.

**Results:**

We identified three groups of homogeneous prenatal care trajectories: (i) women with relatively high prenatal care consumption (~11 %), (ii) women with no prenatal care (~21 %), and (iii) women with an intermediate level of prenatal care (~66 %). Analysis of the socio-economic data demonstrated the association between disparities in prenatal care trajectories and the women’s living environment. Women with relatively high care consumption generally lived in socio-economically privileged areas (better education levels, employment status and housing conditions) compared with women with few or no prenatal care.

**Conclusions:**

Although ecological, our approach demonstrates that data from health administrative information systems could be used to describe prenatal care. However, more individual variables and an improvement of the data quality are needed to efficiently monitor the content and timing of prenatal care. Moreover, state sequence analysis, which was used in this context for the first time, proves to be an interesting approach to explore care trajectories. Finally, the integration of heterogeneous sources of data, including contextual information, might help identifying areas that require health promotion actions toward vulnerable populations, such as pregnant women.

**Electronic supplementary material:**

The online version of this article (doi:10.1186/s12913-015-0857-5) contains supplementary material, which is available to authorized users.

## Background

Understanding care trajectories is crucial for efficient healthcare planning and fair allocation of health care resources [1]. Care trajectories can be defined at the level of healthcare organization (for instance, in France, care pathways are coordinated by the general practitioner) or at the level of individual patients (who can choose to follow or not a given medical advice). Health administrative information systems, which were originally developed for economical purposes, have become useful tools for exploring and understanding care trajectories, particularly when assessing patients’ safety and security [2]. For instance, hospital discharge databases have been used to compute epidemiologic indicators [3] and to monitor the patients’ flow between hospitals [1, 4, 5]. More recent studies have investigated the health reimbursement claims coupled with the hospital discharge records to determine the complete care trajectory of specific patients’ populations [6–8]. Ideally, care trajectory surveillance should include both ambulatory and hospital care. In France, the Programme de Médicalisation des Systèmes d’Information (PMSI) is the national hospital discharge information system that allows monitoring care trajectories during hospitalization, while the Système National d’Informations Inter Régime de l’Assurance Maladie (SNIIRAM; national health insurance information system) permits to follow ambulatory care. Together, these information systems should allow the determination of complete care trajectories.

Here, we investigated whether data from the French national health insurance and hospital discharge information systems allow mining prenatal care trajectories. We chose to follow pregnant women’s care trajectories because they should be regularly monitored by healthcare professionals during the entire pregnancy. In addition, pregnant women represent a vulnerable population for whom care surveillance is essential, notably to minimize adverse effects to themselves and their progeny. Here, we specifically investigated the disparities of care consumption that may exist among pregnant women without health complications. As several studies showed that access to healthcare is influenced by many determinants and is tightly linked to people’s vulnerability [9–11], we focused on the socio-economic environment of the place of residency (data extracted from the national census database), as a risk factor for care disparities. To our knowledge, this is the first French study that integrates hospital discharge, health insurance and national census data to assess disparities in prenatal care consumption.

Moreover, currently care trajectory mining is based on classic statistical methods. Here we took advantage of the sequential data analysis methods described by Gabadinho et al. [12] to dissect care trajectories during pregnancy and identify groups of women with homogeneous care trajectories using clustering analysis. We then used a logistic regression model to describe and characterize each group in relation with their socio-economic environment.

## Methods

### Data

The study sample was composed of women who gave birth in 2009. Ambulatory care data were extracted from the Echantillon Généraliste des Bénéficiaires (or EGB, a permanent sample of the French national health insurance information system SNIIRAM). The EGB is a sample that represents 1/97^th^ of the French population covered by the general health insurance information system. It is representative in term of age and sex, but does not include soldiers, students and government employees. Hospital discharge data were extracted from the Programme de Médicalisation des Systèmes d’Information (PMSI) (now merged with the EGB). We focused specifically on women with uncomplicated pregnancy and delivery, based on their specific childbirth diagnosis that was recorded in the hospital discharge database (PMSI) according to the Tenth Revision of the International Classification of Diseases (ICD10). We selected the codes going from O80.0 to O80.4 and the O30 category (see Additional file 1 for details). In addition, we excluded women who had an abortion or a miscarriage the year before (ICD10 codes going from O00 to O07) because they could have had a tighter antenatal surveillance compared with other pregnant women. From the PMSI, we also extracted the gestational age at childbirth. From the EGB, we selected all the individual variables that characterize pregnant women (age, insurance, government financial support, geographic code) along with the variables that allowed describing the consultations with specific healthcare professionals (date in day/month/year, prescriber, executant, intervention; see Additional file 1 for details). A visit was defined as a consultation for prenatal ultrasound, an obstetric intervention, or any intervention performed by a midwife (including childbirth preparation). Ultrasound examinations could be performed by a general practitioner (GP), a gynecologist, an obstetrician, an imaging center, or a midwife. An obstetric intervention could only be done by a GP, a gynecologist, an obstetrician, or a midwife. All other consultations and other healthcare professionals were not taken into account. EGB and PMSI data were obtained after ethical approval by the Institut des Données de Santé (Approval number 50, received on November 5, 2012).

Socio-economic data were extracted from the 2009 French national census (INSEE). The spatial unit was the municipality (according to the French census definition). The selected variables were associated with the family structure, immigration status, employment, education, and housing (see Additional file 1: Table S2 for details). For comprehensive dissimilarity analyses, socio-economic data were categorized in two groups based on the median of their distribution.

### Time granularity

To estimate the time when women declared their pregnancy to the healthcare authorities, we converted the time of each pregnancy-related visit (recorded in calendar time) into weeks of amenorrhea by subtracting the gestational age at delivery. This measure is relatively prone to noise and might be imprecise. For the care trajectory analysis, we chose to group pregnancy-related visits by trimester because it corresponded to the time periods of potential switch between care professionals. For instance, a woman might change from a GP or a gynecologist to an obstetrician or midwife in the last trimester of pregnancy, when she registers with the hospital where she plans to give birth. Then, the professional allowed supervising a delivery (i.e., obstetrician or midwife) may take over the follow-up at the hospital ambulatory services. A prenatal care trajectory is then defined by the sequence of the number of visits per trimester.

### Continuity of care index

In France, a pregnancy can be monitored by GPs, gynecologists, obstetricians and midwives. To evaluate whether pregnant women change healthcare provider(s) during pregnancy, we computed the continuity of care index (COCI) according to Equation 1 [13]. This index measures the visit dispersion by quantifying the number or percentage of visits to distinct providers.

1 $$ COCI = \frac{{\displaystyle {\sum}_{j=1}^M}{n}_j^2-N}{N\left(N-1\right)} $$

Where N = total number of visits

n_j_ = number of visits to the j^th^ different provider, j = 1, 2… M

M = number of potentially available providers

For visual comparison, the relative frequencies of each healthcare provider’s contribution during the three pregnancy trimesters were also provided.

### Mining sequence data

All computational and statistical analyses were performed using R (Version 3.1.0-R Core Team, 2013). Sequence analysis was performed using the TraMineR library (Version 1.8-8) [12].

For each pregnant woman, visits (as defined above) were transformed into an ordered sequence of states (i.e., trajectory of care). To this aim, the number of visits per trimester of pregnancy was counted and converted into a qualitative variable relative to the quartile distribution of the number of visits. Women with no visits were labeled as having “Absence” of care. Women between the 25 % and 75 % percentiles of the distribution (except those with absence of care) were assigned to the “Intermediate” level of care. And women within the upper quartile (75 % percentile) of the distribution were assigned to the “High” level of care (see Additional file 1: Table S3 for details). Similarities between each woman’s state sequences were computed using the optimal matching (OM) distance algorithm implemented in TraMineR [12]. The OM measure allows time warping for comparing sequences that group pregnant women with a similar level of care. Indeed, we considered that, whatever the trimester of pregnancy, the level of care weighted the same. For instance, a woman with a level of care sequence of the type “Absence-Intermediate-Absence” could be in the same cluster as a woman with the sequence “Intermediate-Absence-Absence”. The substitution-cost matrix used transition rates between states, computed from the set of sequences.

Homogeneous groups of trajectories (clusters) were then built from the distance matrix using agglomerative nesting hierarchical clustering and the Ward’s linkage method. The chosen partition was based on the distances between merging clusters, more specifically we chose the partition with the highest relative loss of inertia (see Additional file 1 for details). The partition quality was assessed using the average silhouette width (ASW) implemented in the WeightedCluster R library [14]. This measure ranges from-1 to 1 and can be interpreted as the coherence of assignments to clusters: high coherence (close to 1) indicates high between-group distances and strong within-group homogeneity.

The analysis of homogeneous groups of care trajectories was based on the women’s individual characteristics and the socio-economic environment of their municipality of residence. Individual characteristics were assessed by univariate analysis using the ANOVA statistical test or Kruskal-Wallis rank sum test and Fisher’s exact test (depending on the variable quality or distribution). A *p*-value threshold of 5 % was applied to all statistical tests for significant differences. For the socio-economic environment data, all women gathered in one homogeneous group of care trajectories (cluster) were compared to all the other women (whatever their group) using a logistic regression model. Before logistic regression modeling, univariate analyses between the outcome (participation to a cluster) and each variable were independently performed using the Fisher’s exact test to select potentially interesting variables to include in the logistic model. Variables with a *p*-value lower than 25 % in univariate analyses were included in the logistic regression model. A manually step-down procedure was used to select the best model for each cluster. A *p*-value threshold of 5 % was applied for significant differences.

## Results

### Population’s characteristics

We analyzed 2518 pregnant women with a mean age of 29.55 ± 5.19 years (range: 14 to 45 years). In France, pregnancy should be declared before the 15^th^ week of amenorrhea. On average the timing of declaration was 14.64 ± 6.72 weeks of amenorrhea (3.29 ± 1.52 in months); 25 women declared after the 35^th^ week of amenorrhea and 28 (1.11 %) did not declare their pregnancy (or their insurance status was not properly completed). These results could be surprising, but they are based on estimations and are prone of noise (see Methods). On average, the gestational age at delivery was 40 ± 2 weeks of amenorrhea.

According to our definition, 140 women (5.55 % of the total population of 2518) did not have any specific consultation during pregnancy; 19 of the 28 women who did not declare their pregnancy were in this group). In addition, 359 women (14 %) had requested at least once to benefit from the so-called couverture maladie universelle et complémentaire (CMUC; universal complementary healthcare coverage).

### Coordinated prenatal care trajectory and continuity of care

**Fig. 1:**
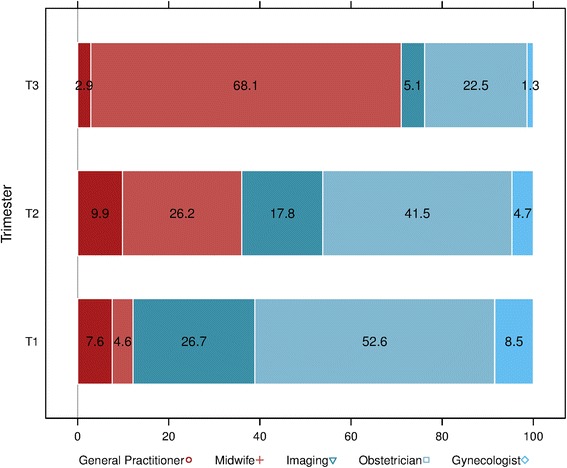
Shift between ambulatory healthcare providers during pregnancy. In France, visits to physicians and imaging centers are predominant during the first two trimesters and then they decrease in favor of midwives’ interventions at the end of pregnancy

A woman followed during her pregnancy had on average slightly more than one visit per month during the first five months of pregnancy and then two-three visits/month till delivery. The increase in the number of visits during the last trimester of pregnancy was mainly due the accumulation of visits to midwives, probably to prepare childbirth. To investigate whether the care quality and efficiency are affected by the coordination of care within and between healthcare organizations and the interaction among healthcare professionals [5, 15], we computed the COCI. The continuity of care during pregnancy was rather low as indicated by the mean COCI of 43.13 % (median 36.4 %) [13] and by the changes in the relative frequencies of the visits to each care provider during pregnancy (Fig. 1). During the first two trimesters, most consultations were with obstetricians (53 % and 42 %, respectively). During the same period, imaging centers (prenatal ultrasounds) also contributed to the follow-up of pregnant women (27 % and 18 %, respectively). Overall, GPs and gynecologists’ interventions were less frequent and decreased progressively during the entire pregnancy, while the midwives’ activities increased significantly over time. During the last trimester of pregnancy, midwives were in charge of most of the follow-up (68 %), whereas the obstetricians’ contribution diminished importantly (down to 23 % from almost 53 % during the first trimester). This is coherent with the fact that, in France, during the last trimester of pregnancy, GPs and gynecologists tend to transfer the surveillance to specialists qualified to supervise delivery (i.e., obstetricians or midwives). Moreover, the high level of midwives’ activities could be in part due to childbirth preparation sessions. The available data did not allow us to distinguish between preparation sessions and follow-up visits by midwives. In addition, some extreme medical practices could be suspected. For instance, 1 % of the women under study had more than nine ultrasounds and up to 16, without any particular underlying medical reason.

### Heterogeneous prenatal care trajectories

**Fig. 2:**
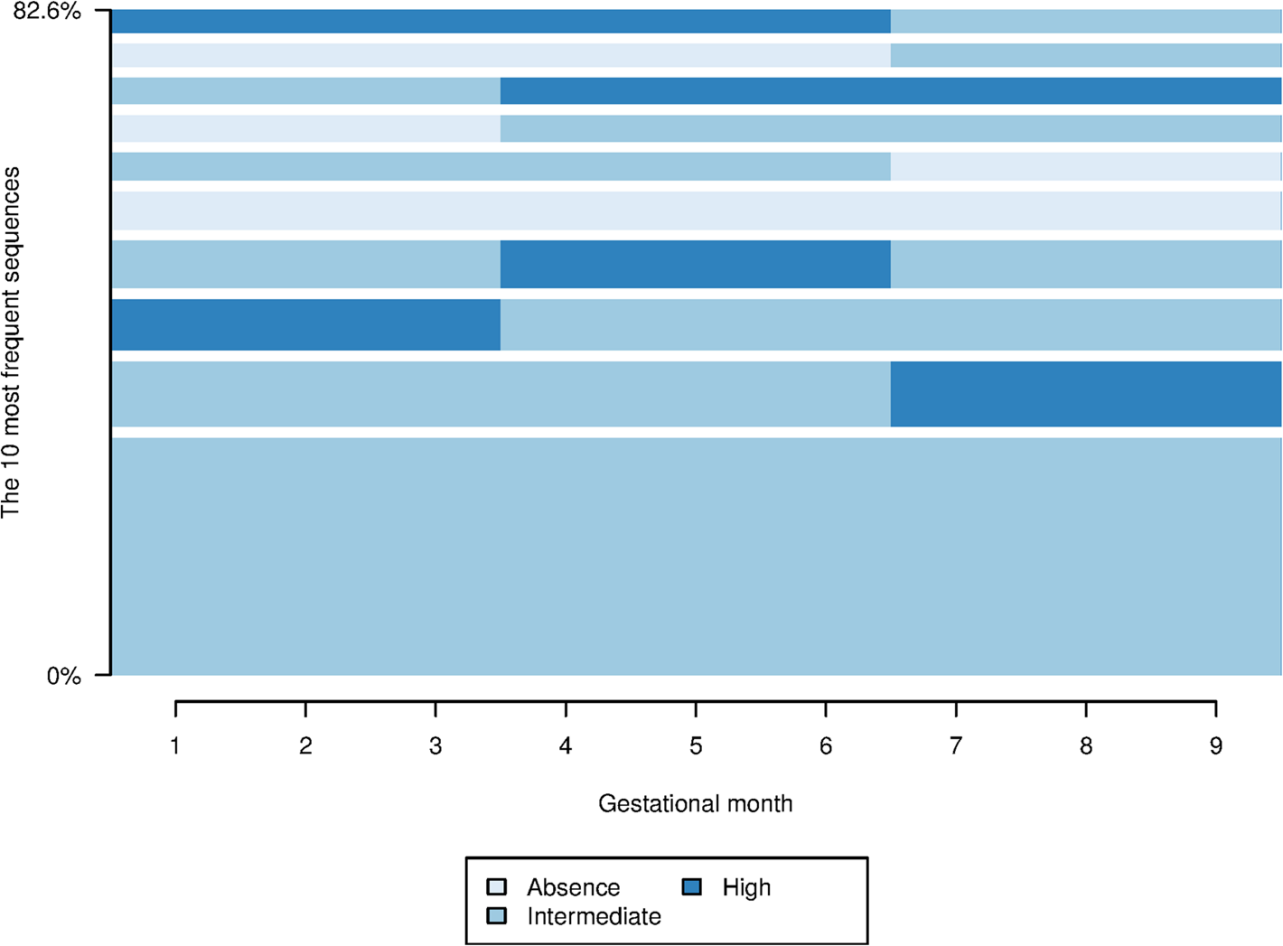
The ten most frequent patterns of antenatal care trajectories. The height of the horizontal bar represents the proportion of pregnant women in the sequence. Overall, the ten most frequent patterns in antenatal care represent 82.6 % of the studied population. The blue colors represent the level of care. Light blue segments are for “Absence” of care, medium blue are for intermediate level, and dark blue are for high level of care consumption

During pregnancy, the number of visits per trimester varied significantly among women and for each woman (Fig. 2). For instance, a woman could have had no visit during the first two trimesters and then many visits during the last one; or many visits during the first trimester, and then few or no visits during the last two. To summarize these frequencies, we categorized the level of prenatal care per trimester according to the quartile distribution of the number of visits (see Methods and Additional file 1 for details). Overall, during the first and second trimester, 16 % of women had no visit and 1 % had six or more visits (max. 16). In the last trimester, 52 % of women had only two visits and 30 % had five or more. If we consider that childbirth preparation might be included in the last trimester counts, the frequency of third trimester visits is quite low (eight sessions of childbirth preparation per woman are fully reimbursed by social security– see Additional file 1 for details). Again, 16 % of women had no visit during the last three months of pregnancy, which is really high. However, these observations do not show whether a woman had a consistent care seeking behavior or was well guided during her pregnancy. The categorization of the data gave 27 distinct sequences. All configurations existed, thus demonstrating the heterogeneity of care trajectories. The most frequent sequence was “Intermediate-Intermediate-Intermediate” and concerned 873 women (34.7 %). The least represented patterns were “Absence-Absence-Intermediate” and “High-High-Intermediate” (84 women/each; 3.3 %). The ten most frequent patterns characterized 82.6 % of the patients (Fig. 2), suggesting that groups of homogeneous (similar) care trajectories existed.

### Homogeneous groups of care trajectories

**Fig. 3:**
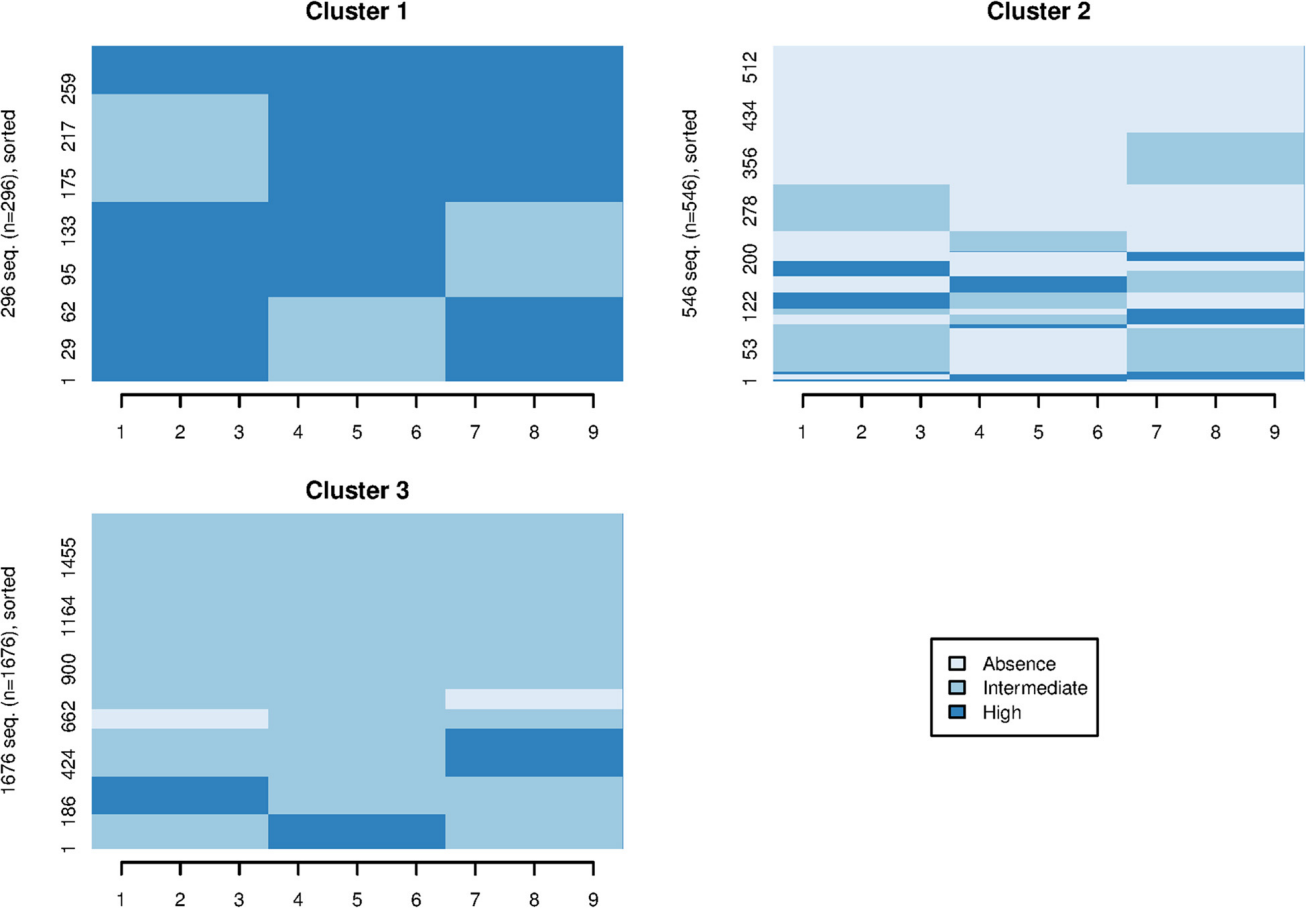
Homogeneous groups of prenatal care trajectories. Each panel represents a group of homogeneous trajectories computed based on clustering analysis. Each cluster includes women with similar sequences of care trajectories (representing specific visits during pregnancy). Cluster 1 gathers women with mostly high level of care consumption. Cluster 2 includes women with low or absence of care. Cluster 3 includes women with mostly intermediate level of care. Each horizontal line (Y-axis) represents a pregnant woman. The X-axis represents the gestational month

Homogeneous groups of care trajectories were computed using clustering methods (see Methods for details). The inertia analysis of the partitioning algorithm suggested three clusters (Additional file 1: Figure S1). The average silhouette width value (ASW = 0.52) used to assess the quality of the partition demonstrated that the clusters had an overall reasonable structure. We obtained similar results within clusters, except for cluster 2 that had an ASW value of 0.32 (see Additional file 1 for other measures). This can probably be explained by the increased diversity of sequences within cluster 2 compared to the other two clusters (Fig. 3). Cluster 1, 2 and 3 included 296, 546 and 1676 women, respectively. Cluster 3 gathered women with mainly an “Intermediate” level of visits, although sub-groups could be distinguished within this cluster. For instance, some women did not consume any care at the beginning or at the end of the pregnancy, while others had a high level of care consumption. Overall, cluster 1 included women who had an elevated number of ultrasound examinations and many midwife’s visits. Cluster 2 was peculiar as 61 % of the women did not have any prenatal care consumption (“Absence”) for at least two full trimesters, although more heterogeneity was visible between sequences than in the other two clusters.

### Socio-economic disparities and prenatal care trajectories

To investigate the determinants of such clustering and disparities of care trajectories, we modeled the influence of women’s characteristics and specifically their socio-economic environment of living. To help the interpretation, we labeled the clusters according to their most frequent pattern: cluster 1 was named “high level of care”, cluster 2 “absence of care” and cluster 3 “intermediate level of care”. Nevertheless, this is a simplification because each cluster was quite heterogeneous as demonstrated by their ASW value.

Univariate analysis showed that the women’s age was significantly different between cluster 1 (“high level of care”) and cluster 2 (“absence of care”) (Tukey’s test p < 0.01), and between cluster 2 (“absence of care”) and cluster 3 (“intermediate level of care”) (Tukey’s test p < 0.05) (Table 1 and Additional file 1 for details). Women in cluster 2 (“absence of care”) were slightly younger (28.86 ± 5.78 years) than in the two other clusters (30.03 ± 4.71 and 29.55 ± 5.08 year for cluster 1 and 3, respectively). The COCI was not significantly different in the three clusters (Kruskal-Wallis rank sum test p = 0.40). Similarly, no difference was detected between clusters concerning CMUC (Fisher’s exact test p = 0.29).

**Table 1:** Univariate analysis describing how each cluster is related to individual characteristics of the population under study

Clusters	“High” level	“Absence” level	“Intermediate” level	*p*-value
Variables	(Cluster 1)	(Cluster 2)	(Cluster 3)	
	N = 296	N = 546	N = 1676	
Age (± sd) in years	30.03 (4.71)	28.86 (5.78)	29.55 (+/−5.08)	0.003^*^
CMUC (%)				
Yes	40 (13.5)	89 (16.4)	230 (13.8)	0.29^**^
No	256 (86.5)	454 (83.6)	1441 (86.2)	
COCI	0.37	0.42	0.40	0.409^***^

*: *p*-value of the ANOVA; **: *p*-value of the Fisher’s exact test; ***: *p*-value of the Kruskal-Wallis rank sum test

Unfortunately, apart from the CMUC, individual data on the women’s socio-economic status were not directly available. We thus used socio-economic data extracted from the women’s place of residency (municipality) to characterize their living environment. We focused on education level, employment status, population type and housing environment (see Additional file 1 for details).

Univariate analysis indicated that many socio-economic variables were related to cluster membership (Table 2). The most significant were education level and employment status. Education level differentiated cluster 1 (“high level of care”) from cluster 2 (“absence of care”). Women in cluster 1 (“high level of care”) were less likely to live in an environment with people without any diploma, whereas it was the contrary for women in cluster 2 (“absence of care”). The employment status discriminated cluster 2 (“absence of care”) from cluster 3 (“Intermediate level of care”). While precarious jobs and unemployment status were risk factors for women in cluster 2, stable jobs were more frequent in cluster 3.

**Table 2:** Results of the univariate analysis to select variables of interest for logistic regression modeling

Variables	OR [IC95%]		
	“High” level	“Absence” level	“Intermediate” level
	(Clusters 1 vs 2-3)	(Clusters 2 vs 1-3)	(Clusters 3 vs 1-2)
	N = 296	N = 546	N = 1676
Age			
14-20 year/old	0.23 [0.1-0.58]**	2.26 [1.6-3.2]***	0.74 [0.53-1.03]****
21-35 year/old	1	1	1
>35 year/old	NS	1.29 [0.99-1.68]****	0.84 [0.67-1.07]****
Population type^a^			
Single women	NS	1.34 [1.10-1.63]**	NS
Single mums	0.77 [0.60-0.99]*	NS	0.82 [0.69-0.97]*
Immigrants	0.84 [0.73-1.20]****	NS	NS
Education			
2 years after high school	1.23 [0.96-1.59]****	0.78 [0.64-0.95]*	NS
University	1.16 [0.68-1.13]****	NS	NS
No Diploma	0.63 [0.49-0.82]***	1.24 [1.02-1.51]*	NS
Technical Education	NS	NS	0.87 [0.79-1.03]****
Employment			
Unemployed	NS	1.41 [1.16-1.71]***	0.79 [0.66-0.93]**
Self-employed	1.36 [1.05-1.75]*	NS	0.88 [0.74-1.04]****
Precarious job	1.18 [0.92-1.52]****	1.29 [1.06-1.57]**	0.75 [0.63-0.89]**
Stable job	NS	0.75 [0.61-0.91]**	1.32 [1.12-1.57]***
Labor force	NS	0.72 [0.59-0.88]**	1.28 [1.08-1.57]**
Blue-collar	NS	1.24 [1.03-1.52]*	0.89 [0.75-1.06]****
White-collar	NS	0.84 [0.69-1.02]****	NS
Artisan	1.29 [1.01-1.67]*	NS	0.87 [0.73-1.03]****
Housing			
House built before 1949	NS	1.19 [0.97-1.44]****	NS
House built after 1999	1.49 [1.16-1.92]**	0.78 [0.64-0.95]*	NS
House	NS	NS	NS
Apartment	NS	NS	NS
Rent	NS	1.14 [0.94-1.39]****	NS
Low rent	0.73 [0.57-0.94]*	NS	NS

***p-value <0.001; **p-value <0.01; *p-value <0.05; ****p-value <0.25; NS: not selected *p*-value >0.25

a: Single mums: single mother per household; Single women: woman living on her own per household

When variables were adjusted against each other in logistic regression models, only a few remained significantly associated with cluster membership. Women in cluster 2 (“absence of care”) were 2.17 times more likely to be younger than 21 and 1.32 times more likely to be older than 35. Women in cluster 1 (“high level of care”) were approximately four times less likely to belong to the 14–20 year/old category than to the 21–35 year/old reference category. However, in this cluster, no significant difference was observed between women of the reference category and women older than 35 years. Similarly, no statistical difference in age was observed in cluster 3 (“intermediate level of care”). Concerning the employment status, women with “absence” of care consumption (cluster 2) were 1.27 times more likely to live in municipalities with a level of unemployment above the median, compared with women of cluster 1 and 3. Women with an “intermediate level of care” (cluster 3) were approximately 25 % less likely to live in municipalities with a level of unemployment above the median compared with women in cluster 1 and 2. Additionally, women with absence or low level of care (cluster 2) were 1.42 times more likely to live in municipalities with a proportion of blue-collars above the median than women in cluster 1 and 3. Women in cluster 3 were also less likely to live in municipalities with levels of precarious jobs and proportion of artisans above the median. The education level was more complex to analyze. No education variable was significant in the “absence of care” cluster (cluster 2). However, women in cluster 1 (“high level of care”) were less likely to live in municipalities with inhabitants without a diploma than women in the other two clusters. Concerning accommodation, women in cluster 1 (“high level of care”) were 33 % more likely to live in cities with more recent buildings (i.e., built after 1999).

## Discussion

Our analysis highlights the overall heterogeneity of care trajectories among pregnant women, especially regarding ultrasound follow-up, in France. Nevertheless, three groups of homogeneous care trajectories could be identified: (1) pregnant women with relatively high care consumption (cluster 1; 11 % of the population under study), (2) women with no care (cluster 2; 21 %), and (3) women with an intermediate level of care (cluster 3; 66 %). These findings slightly differ from the prospective observational study by Beeckman and colleagues showing that in Belgium only 10-18 % of women had inadequate to intermediate antenatal care trajectory, whereas 64 % had sufficient to appropriate level of care [11]. However, these authors could investigate the content and timing of antenatal care, whereas we could focus only on the number of visits to healthcare providers involved in antenatal care. On the other hand, in our retrospective study based on data extracted from information systems, the analysis of antenatal care trajectories was free from potential response bias (i.e., women who are aware of being part of an experiment might behave differently). The main drawback of the databases used in our study was the lack of individual information to precisely characterize our patients and explain their inclusion in one of three groups of homogeneous care trajectories. Among the few available individual variables (age, COCI and CMUC), only age was significantly associated with belonging to a specific cluster. Surprisingly, women with insufficient or absent level of antenatal care were more likely to be in the youngest or the oldest age category (Table 3), despite the current recommendations concerning increased surveillance for those ages and especially for late pregnancies.

**Table 3:** Importance of education and employment status for pregnant women care trajectories

Variables	OR [IC95%]		
	“High” level	“Absence” level	“Intermediate” level
	(Clusters 1 vs 2-3)	(Clusters 2 vs 1-3)	(Clusters 3 vs 1-2)
	N = 296	N = 546	N = 1676
Age			
14-20 year/old	0.25 [0.1-0.61]**	2.17 [1.53-3.08]***	NS
21-35 year/old	1	1	1
>35 year/old	NS	1.32 [1.01-1.72]*	NS
Population type			
Single women	NS	1.37 [1.10-1.70]**	0.82 [0.68-0.99]*
Education			
No Diploma	0.71 [0.54-0.92]**	NS	NS
Technical Education	NS	NS	0.81 [0.67-0.97]*
Employment			
Unemployed	NS	1.27 [1.03-1.55]*	0.76 [0.63-0.93]**
Blue-collar	NS	1.42 [1.15-1.73]***	NS
Precarious job	NS	NS	0.82 [0.69-0.98]*
Artisan	NS	NS	0.79 [0.67-0.97]*
Housing			
House built after 1999	1.34 [1.03-1.73]*	NS	NS

The results of the logistic regression models describe how each cluster membership relates to specific covariates

***p-value <0.001; **p-value <0.01; *p-value <0.05; NS: not significant

To circumvent the lack of individual information, we made use of the socio-economic data from French municipalities. Contextual data on the women’s place of residence represented informative and appropriate alternatives to individual socio-economic characteristics [8, 16]. Although the results are ecological, they are in coherence with other published works. For instance, Blondel and Marshall [9] showed that risk factors for poor attendance were single status (also suggested by our analysis) and lack of health insurance for women younger than 20 years. Similarly, lower education (like in our study) and North African descent were predisposing determinants that explained the higher likelihood of receiving an unfavorable level of antenatal care in pregnancy [11]. The most interesting result of our analysis is the effect of the employment status, suggesting that public health messages addressed to pregnant women should be given at the workplace to maximize their impact.

Nevertheless, our results should be taken with caution. Indeed they are difficult to generalize to the entire French population of pregnant women because they are based on data extracted from a sample (1/97^th^) of the French national health insurance information system that does not include military people, students and government employees. Moreover, due to territorial disparities in birth rate, some geographical areas were under-represented, thus decreasing the power of our conclusions. It would be interesting to require access to the entire French national health insurance information system to perform some multilevel analyses. Moreover, in future studies the reference antenatal care trajectory should be more precisely defined by integrating all visits and blood/urine testing recommended by the health authorities. This would give a more accurate picture of what is an adequate follow-up of pregnant women and would improve the quantification of how many women deviate from the guidelines. The quality of the data recorded in the information system should also be improved; indeed, the lack of individual information did not allow determining whether antenatal care consumption was associated with the women’s care seeking behavior or with the facility of access to care. Future studies should also focus on specific French districts and conduct qualitative post-partum interviews with women and their healthcare providers. This could help (i) refining the homogeneous groups of care trajectories and (ii) assessing the socio-economic determinants that lead pregnant women to have too few or too many visits.

## Conclusions

Integrating healthcare data with contextual (spatial) information might help identifying areas that requires health promotion actions towards vulnerable populations, such as pregnant women. Our pilot study demonstrates that mining the prenatal care trajectories of pregnant women using the French health insurance information systems together with socio-economic contextual data is feasible, but complex due to the data heterogeneity and quality. Although the primary purposes of administrative medical information systems are financial and managerial, we show that they can give interesting insights for care trajectories analysis. They could provide tools for epidemiological surveillance and might be used for evaluating healthcare policies. Moreover, state sequence analysis with adapted identification algorithms and the associated statistical analyses are interesting approaches that could be applied to investigate other health issues, such as chronic disease conditions.

## Additional file

Additional file 1:
**Mining care trajectories using health administrative information systems: the use of state sequence analysis to assess disparities in prenatal care consumption.**

## Abbreviations

ASW: Average silhouette width
EGB: Echantillon Généralistes des Bénéficiaires
SNIIRAM: Système National d’Informations Inter-Régime de l’Assurance Maladie
PMSI: Programme de Médicalisation des Systèmes d’Information
ICD10: International statistical classification of diseases and related health problems 10th revision
INSEE: Institut National de la Statistique et des Etudes Economiques
ANOVA: Analysis of variance
COCI: Continuity of care index
CMUC: Couverture maladie universelle et complémentaire or universal complementary healthcare coverage.
