# Supplementary material for: Mining care trajectories using health administrative information systems: the use of state sequence analysis to assess disparities in prenatal care consumption
Source: BMC Health Serv Res. 2015 May 15;15:200. doi: 10.1186/s12913-015-0857-5 (PMC4436876; doi:10.1186/s12913-015-0857-5)
Supplement: Additional file 1: — Mining care trajectories using health administrative information systems: the use of state sequence analysis to assess disparities in prenatal care consumption. [file 12913_2015_857_MOESM1_ESM.docx]

**Mining care trajectories using health administrative information systems: the use of state sequence analysis to assess disparities in prenatal care consumption**

**Supplementary material**

Nolwenn Le Meur*^1,2^, Fei Gao^1,3^, Sahar Bayat^1,2^

^1^ Department of Epidemiology and Biostatistics, French School of Public Health (EHESP), Sorbonne Paris Cite, France

^2^ EHESP, EA 7348 MOS Management des organisations en santé, France

^3^ Inserm, UMR IRSET Institut de recherche sur la santé l’environnement et le travail - 1085, France

* Corresponding author

Department of Epidemiology and Biostatistique Ecole des Hautes Etudes en Sante publique Avenue du Professeur Leon Bernard 35043 Rennes France

E-mail: nolwenn.lemeur@ehesp.fr

Phone: +33 2 99 02 25 14

# Methods

## Data from the hospital discharge information system (PMSI)

Below are listed the categories of the International Classification of Diseases selected to identify women whose pregnancy and delivery went without complications.

***O30 Multiple gestation***

O30.0 Twin pregnancy

O30.1 Triplet pregnancy

O30.2 Quadruplet pregnancy

O30.8 Other multiple gestation

O30.9 Multiple gestation unspecified

***O80–O84 Delivery***

O80 Single spontaneous delivery

O80.1 Spontaneous breech delivery

O81 Single delivery by forceps and vacuum extractor

O81.4 Vacuum extractor delivery

O82 Single delivery by caesarean section

O83 Other assisted single delivery

O84 Multiple delivery

In addition we removed women who had an abortion or a miscarriage (ICD10 codes from O00 to O07) the year before their current pregnancy as they might have been under higher surveillance than normal. We also removed premature births (gestational age < 37 weeks) and one woman who had two pregnancies during our period of analysis.

## Data from the health insurance information system (EGB)

The EGB provides the ambulatory care information. The variables used are listed below.

**Variable PSE_SPE_COD** refers to the type of healthcare provider.

We chose the following codes as they represent healthcare providers who might be involved in prenatal care:

Code 7: obstetrician

Code 77: gynecologist obstetrician

Code 79: gynecologist obstetrician and general gynecologist; recoded Code 77 because they have obstetric qualifications.

Code 70: gynecologist (i.e., cannot supervise delivery)

Code 1: general practitioner

Code 6: imaging laboratories

Code 21: midwife

An obstetrician is a physician who successfully completed specialized education and training in the management of [pregnancy](http://www.pediatrichealthchannel.com/prenatal-health/pregnancy-information.shtml), labor and postpartum period (the period directly after childbirth).

A gynecologist is a physician who successfully completed specialized education and training in the health of the [female reproductive system](http://www.womenshealthchannel.com/menstruation/index.shtml#menstrual-cycle), including the diagnosis and treatment of disorders and diseases. They are not allowed to assist women in giving birth.

**Variable PRS_NAT** refers to the prescribed interventions. We took into account:

Code 1324 labeled ACTE D'ECHOGRAPHIE CCAM (ultrasound examination)

Code 1322 labeled ACTE D'OBSTETRIQUE CCAM (obstetric intervention)

Code 1911 labeled ACTES DES SAGES-FEMMES (midwife’s intervention)

Code 1316 labeled ACTES DE DIAGNOSTIC COTES KE (ultrasound scan performed by a midwife)

**Tableau 1S. Number of pregnancy-related visits performed by the different healthcare professionals under study (number of women investigated N=2518)**

**(Source: EGB, 2008-2009)**

| Specific visits  Care professionals | Ultrasound scan | Obstetric intervention | Midwife’s intervention | Ultrasound (by midwives) |
| --- | --- | --- | --- | --- |
| General physicians | 983 | 1 | 0 | 0 |
| Imaging centers | 2298 | 1 | 0 | 0 |
| Obstetricians | 5176 | 838 | 0 | 0 |
| Gynecologists | 661 | 2 | 0 | 0 |
| Midwives | 6 | 0 | 7989 | 7 |

**Variable RGO_ASU_NAT** refers to the women’s insurance status

Women must declare their pregnancy to their health insurance. In the database this is coded with the RGO_ASU_NAT variable which takes the modality « MATERNITE » (Pregnancy). Sometimes this variable was not correctly updated.

**Variable BEN_CMU_TOP** refers to women’s registered for the universal complementary healthcare coverage (Couverture Maladie Universelle Complémentaire; CMUC). CMUC is a social aid proposed by the French healthcare system to socially deprived individuals.

## Data from the French National Institute of Statistics (INSEE): socio-economic data from the 2009 French national census

Table 2S lists the variables used to compare and explain the results of our analysis. “Single women” are women living by themselves, whereas “single mums” are mothers taking care of their children alone (without a man).

**Table 2S. Variables and definitions of the socio-economic data used as potential covariates for the analysis of prenatal care trajectories (Data from INSEE, 2009)**

| French label | English translation | Definition and units per place of residency |
| --- | --- | --- |
| Code_Geo  Num_anonyme  FMONO  FSEUL  IMMIGRANTS  CHOM  EPREC  ESTA  ACT  NSAL  OUVR  CADR  ARTI  NDIP  CAPBEP  BACP2  SUP  NPROP  HLM  AV1949  AP1999  MAISON  APPART | Code_Geo  Num_anonymous  SingleMum  SingleWoman  Immigrants  Unemployment  Precarious Job    Stable Job  Active  Self-Employed  Blue Collar  White Collar  Artisan  No Diploma  Technical Education  2YearsAfterHigh School  University  Rent  LowRent  Before 1949    After 1999  House  Apartment | Zip code  Anonymous Identification  Single mother per household  Woman living on her own per household  Immigrants per inhabitant  Unemployment per inhabitants (15 to 64 year/old)  Precarious job per active population (15 to 64 year/old)  Stable job per active population (15 to 64 year/old)  Labor force per inhabitants (15 to 64 year/old)  Self-employed per inhabitants (15 to 64 year/old)  Blue collar per inhabitants (15 to 64 year/old)  White collar per inhabitants (15 to 64 year/old)  Artisan, craftsman per inhabitants (15 to 64 year/ old)  Without diploma per graduates aged 15 or more  Technical education per graduates aged 15 or more  Two years after high school per graduates aged 15 or more  University diploma per graduates aged 15 or more  Rent their apartment/home per owners of a residence  Low rent housing per owners of a residence  Accommodation built before 1949 per owners of a residence  Accommodation built after 1999 per owners of a residence  Number of houses per owners of a residence  Number of apartments per owners of a residence |

## Mining sequence data

Table 3S presents the quartile distribution of the number of visits per trimester of pregnancy. Those values were used as thresholds to convert the observed number of visits for each woman into a categorical variable. We defined three levels of care: “Absence”, ”Intermediate” and “High”. As we focused on specific visits (ultrasounds, obstetric and midwife’s interventions), intermediate level of care was defined by a number of visits within the lowest 75% of the distribution. High level of care was defined by a number of visits within the highest 25% of the distribution.

For instance, a woman had 0 then 2 and 4 visits during the first, second and third trimester, respectively. Converted into levels, this woman had an “absence of care” level for the first trimester and then an “intermediate” level of care for the second and third trimester.

Graphically, the sequence would be:

Woman #1

T1-“Absence of care” T2 “Intermediate” T3 “Intermediate”

A second woman (#2) had 0-8-6 visits. This corresponded to “absence of care”-“high”-“intermediate”

Woman #2

T1-“Absence of care” T2 “High” T3 “Intermediate”

**Table 3S. Quartile distribution of the number of visits per trimester of pregnancy (computed from the analysis of EGB 2008-2009 for 2518 women). In grey is highlighted the range of visits considered as intermediate level of care.**

| Period of pregnancy  Categorical variable | Quartile | Trimester 1 | Trimester 2 | Trimester 3 |
| --- | --- | --- | --- | --- |
| Absence of care |  | 0 | 0 | 0 |
| Intermediate | 25% | 1 | 1 | 1 |
|  | 50% | 1 | 1 | 2 |
|  | 75% | 2 | 2 | 7 |
| High (above 75%) | 100% | 16 | 15 | 38 |

We could not assess whether the women actively required care or were guided through the French guidelines for pregnancy care trajectory. For information, we give in the paragraph below an overview of these guidelines.

**French guidelines for pregnancy care trajectory**

A pregnant woman must inform her local healthcare office at least four calendar months prior to the expected delivery date (for reimbursement of the healthcare costs). She then should follow a schedule of pre- and post-natal medical examinations the full cost of which is met by the social security system. 
There are seven obligatory pre-natal examinations that must be undertaken at specific intervals beginning with a first examination at the end of the third month of pregnancy and then every month from the 4^th^ month onwards. Three ultrasound examinations are suggested at month 3, 5 and 8 of pregnancy. Additional laboratory examinations will be proposed to verify immunity against specific diseases (toxoplasmosis hepatitis B, rubella…).

From the beginning of the pregnancy up to the end of the 5th month:

- The costs of the different medical examinations and laboratory analyses included in the surveillance are fully reimbursed

- Specific examinations, such as amniocentesis and karyotype analysis, are also fully reimbursed

- Ultrasounds are 70% reimbursed

From the 6th month of pregnancy up to 12 days after delivery all healthcare costs are 100% reimbursed (including dental care)

Eight sessions of childbirth preparation are also proposed and fully reimbursed. They are usually given by midwives.

# Results

## Clustering analysis

The chosen partition was based on the distances between merging clusters (height of branches in the clustering tree), more specifically we chose the partition with the highest relative loss of inertia.

Figure 1S presents the results of the agglomerative nesting hierarchical clustering with the Ward's method for linkage analysis of the 2518 care trajectories. It suggests a partition in three groups (clusters).


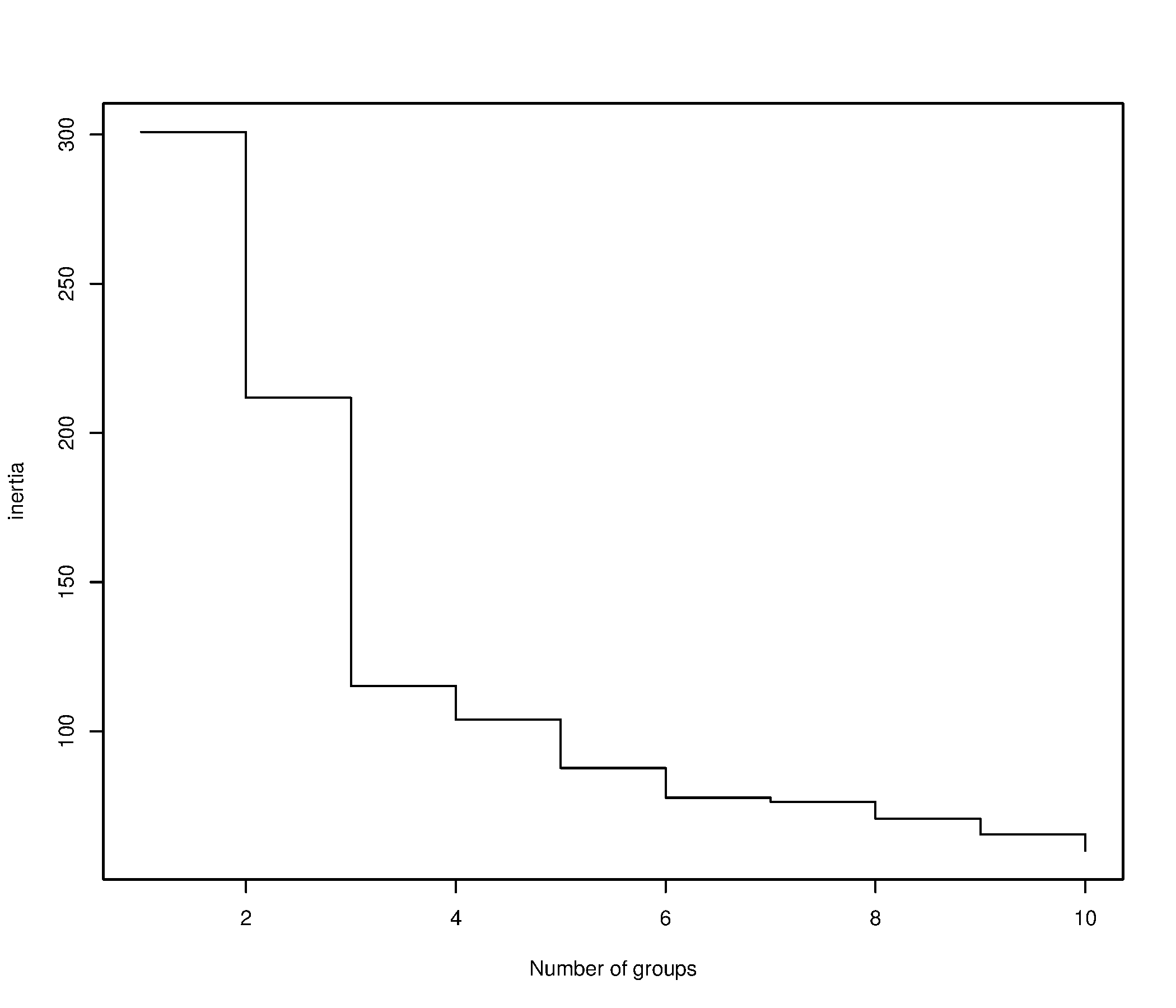


**Figure 1S. Inertia analysis of the agglomerative nesting hierarchical clustering**

We assessed the quality of the partition using the measures proposed by the *WeightedCluster* R library {Studer:2013bv}. The box below displays the results (rounded) of the call to the quality assessment function in R.

> wcClusterQuality(distOM, clust3)

$stats

PBC HG HGSD ASW ASWw CH R2 CHsq R2sq HC

0.74 0.89 0.87 0.52 0.52 910.89 0.42 2303.46 0.65 0.03

$ASW

Cluster ASW ASWw

1 0.57 0.57

2 0.32 0.32

3 0.57 0.57

## Age differences between clusters

We tested whether women in the different clusters had the same mean age. To this aims, we performed an ANOVA (alpha risk 5%). In case of statistical differences, we performed a Tukey’s multiple comparisons of means. The box below presents the results.

> modAge = aov(age~as.factor(cluster), data=medical)

> summary(modAge)

Df Sum Sq Mean Sq F value Pr(>F)

as.factor(cluster) 2 307 153.38 5.665 0.00351 **

Residuals 2507 67883 27.08

---

Signif. codes: 0 ‘***’ 0.001 ‘**’ 0.01 ‘*’ 0.05 ‘.’ 0.1 ‘ ’ 1

> TukeyHSD(modAge)

Tukey multiple comparisons of means

95% family-wise confidence level

Fit: aov(formula = age ~ as.factor(cluster), data = medical)

$`as.factor(cluster)`

diff lwr upr p adj

2-1 -1.1703686 -2.05202356 -0.2887136 0.0053187

3-1 -0.4756478 -1.24518841 0.2938929 0.3157472

3-2 0.6947208 0.09193345 1.2975081 0.0189696
